# Mucous Colitis

**Published:** 1903-04-11

**Authors:** 


### Hospital Clinics and Medical Progress.

### MUCOUS COLITIS.

The disease more generally known as mucous colitis is also described under various other headings as membranous colitis, mucous or membranous enteritis, desquamative enteritis, tubular diarrhœa, mucous colic, etc. Clinically it is characterised by constipation, abdominal pain of a more or less paroxysmal nature, and the passage *per rectum* of gelatinous material in the form of membranes, shreds, or masses. Some concomitant nervous symptoms are also almost invariably present.

The disease is much more common in women than in men. Some authors give the proportion as 4 to 1, but Langenhagen,[1] on analysing 600 cases observed by himself, found that 141 occurred in men, 435 in women, and 24 in children. The majority of the patients are over 20 years of age. The disease appears to be commoner on the Continent and in America than in this country. The patients chiefly affected are of the chronically depressed, dyspeptic, and neurasthenic type.

The disease has been generally described as a secretory neurosis of the large bowel. Langenhagen, as a result of his wide study of cases, lays great stress on the presence of arthritic symptoms in conjunction with a nervous dyscrasia. In 560 of his 600 cases nervous and arthritic symptoms were present, combined in 524, while in 25 cases nervous, and in 11 cases arthritic symptoms alone were observed. In only 40 cases was no trace of "nervous arthritism" discovered, and in many of these infectious illness, especially typhoid, seemed to be the starting point of the disease, whilst in eight cases only did a true neurasthenia exist before the establishment of the gastro-intestinal disorder. Some pre-existing nervous phenomena were noted as having become augmented in 245 cases, whilst in 144 these symptoms developed into one or more definite signs of neurasthenia, such as sleeplessness, headache, depression, inaptitude for intellectual work, etc. Langenhagen quotes his figures as disproving the theory that neurasthenia is the primary cause of the intestinal trouble, although mucous colitis invariably implants itself on those who show a nervous and arthritic predisposition. In such atony of the muscular tissue of the colon predisposes to irritation of the mucosa and hypersecretion of the glands. It is, however, he maintains, equally certain that once established mucous colitis exaggerates or creates the nervous condition which may advance to true neurasthenia under the double influence of reflex action and autointoxication. These views have recently been endorsed by an English writer, T. S. Wilson,[2] who, discussing broadly the subject of colon catarrh, regards mucous colitis merely as one variety of this catarrh not sharply separated from but shading off into the ordinary subacute and chronic forms. He believes in the gouty origin of many cases, following Haig's theory, that occasional excessive accumulations of uric acid in the cæcum may set up a catarrh of that organ, and regards the nervous symptoms as secondary to the depressing effect of the colon irritation.

The whitish brown material passed by the bowel may appear in masses likened to frog's spawn, or it may be rolled into small tight balls or appear as bands or membranes, or as definite tubular casts of the intestine several inches or even a foot or more in length. From their colour and general appearance they have been aptly likened to pieces of tapeworm. The membranes, which consist of mucus, are semi-transparent and structureless. Sometimes they show distinct longitudinal bands, a sign according to one observer of spastic constipation,[3] or they may be sacculated or marked by the valvulæ conniventes, or show minute perforations corresponding to the orifices of the ducts. On floating a tubular portion in water it is seen to be smooth inside and shaggy outside, somewhat resembling in appearance an early gestation sac. Sometimes the membranes are laminated, fæcal matter appearing between the layers, a fact which would seem to suggest that the mucus has been secreted higher up the bowel and has been deposited lower down, whilst imbedded in the structureless mass are found particles of food and of fæces, epithelial cells, putrefactive micro-organisms, crystals of cholesterin, and phosphates. Chemically the material has been frequently found to consist of albumin with no fibrin. Sometimes only a trace of mucin has been detected. Evidence obtained post-mortem does not support the inflammatory origin of the disease sometimes advocated. Osler mentions that he has twice seen the membrane *in situ*. The mucus was closely adherent to the mucosa but was separable without any lesion. Hale White,[4] in a fatal case in a man marked by severe hæmorrhages, found nothing more than a dilated colon with thin atrophied walls and a few patches of congestion. Reddening of the mucosa with denudation of the superficial epithelium has been described.

The symptoms vary considerably in intensity and duration. The typical patient is thin, dyspeptic, and highly "nervous," being subject to much chronic depression and exhibiting a low level of vitality. The extremities are often chilly and the circulation feeble. The tongue is furred and pale and the lips may persistently peel. The bowels are generally obstinately constipated. Rarely constipation is replaced by diarrhœa. Hæmorrhoids are very frequent. Amenorrhœa and pelvic troubles have been frequently described in women, but were present in only 19 per cent. of Langenhagen's cases. Constipation, abdominal pain, and the passage of membranes or masses of mucus are the three cardinal symptoms. The muco-membranous material is not passed continuously. Its passage is preceded by griping abdominal pain, which is often localised in the right iliac fossa, but may be referred to the umbilicus or more generally diffused over the abdomen. Vomiting may occur during the attacks and there may be diarrhœa at these times. In milder cases the attacks may last for a day or a week, and then clear up and not recur for weeks or months, but in the more severe cases the symptoms may persist more or less continuously for weeks or months together. Slight fever may be present and increased resistance and tenderness may be detected in the right iliac fossa. The cases then closely resemble appendicitis, with which they have been confused, though according to some these symptoms with fever mean an associated appendicitis.[5] Mental depression and causeless misery are apt to be very marked during the attacks. Pain is occasionally absent. Wilson points out its liability to occur an hour or so after food and to be brought on by some exertion, such as walking. Occasionally the membranes or "skins" may be tinged with blood, and in severe cases bright-red blood may be passed per rectum. Urticaria has been seen, and also superficial ulceration of the mouth.[6] Langenhagen describes other symptoms, such as gastric atony and consequent dilatation, and relaxation of the abdominal ligaments leading to enteroptosis and ptoses of the other viscera occurring simultaneously with or preceding the intestinal atony. Dilatation of the cæcum, often marked, is also described. Wasting may be present, and reflex nervous troubles, such as palpitation, vertigo, dyspnœa, sweating, etc. Both he and Mathieu[7] have noticed a reduction in the size of the liver, which they think may account for the paleness of the fæces sometimes observed independently of the occurrence of jaundice. A case at present under observation typically presents many of these characteristics. The patient, a tailoress aged 34, is thin, weakly, dyspeptic, breathless and neuralgic, never robust, the subject of hæmorrhoids and of a retroversion. She first came under observation for an obscure affection of the right hip simulating mild tuberculous disease. After a period of generally feeble health advice was sought for abdominal pain, which had been more or less present for some months. Slight fever, vomiting, constipation and severe abdominal pain, with tenderness and swelling localised in the right iliac fossa, suggested inflammation about the appendix until, after some weeks, the passage of typical pieces of membrane led to a revision of the diagnosis. Slight fever persisted during a constant observation extending over three or four months, and twice fairly free recurrent hæmorrhages have occurred due to no discoverable lesion in the rectum, the hæmorrhoids having been removed by operation. Abdominal pain of greater or less severity and the occasional passage of "skins" have persisted for some months. Constipation, although generally present, has not been a very marked feature in this case. Microscopically the membrane showed the usual structureless appearance described, but the reaction for albumin was weak or absent, while the material dissolved readily in strong caustic potash.

Mild cases recover under suitable treatment, more severe cases are intractable and apt to lead to chronic invalidism. Death has occurred from exhaustion, and also in some cases attended by severe hæmorrhage. In the matter of treatment all observers are agreed as to the harmfulness of purgatives or even of laxatives, except in the very mildest cases. The bowels should be kept open by warm water or soap and water enemata, or by small injections of oil. Food should be light, nourishing, and easily digested; substances such as brown bread, and green vegetables leaving a bulky or irritating residue, being avoided. Tonics, change of air, and hydrotherapy are valuable for the nervous condition. Wilson strongly advises the salicylates, or some combination of these with phenol, such as salol. Flushing the colon under *gentle* pressure, using a soft rubber tube, has proved very effective in many cases. Saline solution, or a weak solution of boracic acid, may be used. Mathieu advises the addition of neutral ichthyolate of ammonia. Belladonna, cannabis indica, ammonium valerianate, and the perchlorides of iron and mercury in combination have also been recommended. In desperate cases right inguinal colotomy has been tried successfully, the wound being kept open for many months in order to give the colon rest.

[1] Trans. International Cong. of Med., 1900, Vol. IV., p. 110. [2] Brit. Med. Jour., Dec. 6, 1902. [3] Westphalen Prog. Med., Vol. IV., 1901, p. 62. [4] Allbutt's System of Medicine. Vol. III., p. 943. [5] Gibson's System of Medicine, Vol. I., p. 714. [6] Practitioner, Vol. I., 1893. [7] Trans. International Med. Cong., 1900, Vol. IV., p. 101.

